# PROTECTIVE-D—a feasibility randomised controlled trial examining the effect of a positive mood intervention and vitamin D supplementation on the immunological responses to influenza vaccination in older adults

**DOI:** 10.1186/s40814-026-01881-z

**Published:** 2026-07-14

**Authors:** Simon Royal, Kieran Ayling, Georgina Hopkins, Lucy C. Fairclough, Kavita Vedhara

**Affiliations:** 1 University of Nottingham Health Service, Cripps Health Centre, Nottingham, UK; 2 Centre for Academic Primary Care, School of Medicine, University of Nottingham, Nottingham, UK; 3 School of Life Sciences, University of Nottingham, Nottingham, UK; 4 School of Psychology, Cardiff University, Cardiff, UK

**Keywords:** Influenza vaccination, Positive mood, Vitamin D supplementation, Vaccine effectiveness, Older people

## Abstract

**Background:**

The effectiveness of vaccines in protecting against disease wanes with increasing age. There is some evidence that psychological wellness and vitamin D supplementation can increase vaccine effectiveness in older people. This study was designed to pilot the acceptability of, and compliance with, study procedures intended for a future large randomised controlled trial (RCT). The procedures included a positive mood intervention (PMI), the use of vitamin D supplements and self-collection of finger-prick blood samples.

**Methods:**

Twenty-eight adults aged > 65 years were randomised to one of four conditions: (1) PMI and vitamin D supplements, (2) PMI only, (3) Vitamin D supplements only and (4) Usual care. Blood samples were taken before vaccination with the standard seasonal influenza vaccine and again after 28 days. Vitamin D levels and antibodies to influenza were compared before and after the participants received the allocated intervention.

**Results:**

Good compliance with study procedures, as measured by participant responses to a Short Message Service (SMS) text or email, suggest methods were acceptable and feasible in this population. Self-collection of finger-prick samples to measure antibody responses was accomplished by 16 out of 26 participants (62%).

**Conclusions:**

This study demonstrates that a large-scale RCT to evaluate two interventions promoting vaccine effectiveness in older adults is feasible. Simple methods of determining patient-reported outcomes and limiting study visits are acceptable to participants and may reduce costs. The finger-prick capillary sampling procedure requires refinement before wider deployment.

**Trial registration:**

ISRCTN 12950625 08 FEB 2024 (registered retrospectively).

**Supplementary Information:**

The online version contains supplementary material available at https://doi.org/10.1186/s40814-026-01881-z.

## Key messages regarding feasibility

- What uncertainties existed regarding the feasibility?
  This feasibility study was designed to determine whether the methods proposed for a large RCT were suitable in an older population. It may be assumed that older people will have difficulty using technology and performing study procedures, such as remembering to take medication and collecting samples, and this can have implications for study design and cost.
- What are the key feasibility findings?
  This study has demonstrated that the interventions were acceptable to the proposed study population and that participant-reported outcomes related to compliance with study procedures can be effectively monitored using responses to electronic communications. It is likely that self-collection of finger-prick samples will be feasible in this population, although incorrect filling of the tubes in some cases suggests further refinement of the procedure is necessary before wider deployment.
- What are the implications of the feasibility findings for the design of the main study?
  The use of electronic communications and/or e-diaries in the proposed main study is supported. The use of self-collected finger-prick samples is likely to be feasible in this population, although additional work is necessary to develop means of supporting those participants who lack confidence or manual dexterity.

## Introduction

### Background

Vaccines play a critical role in reducing the burden of infectious disease in the population. An estimated 154 million lives have been saved since the introduction of the WHO Expanded Programme on Immunization in 1974 [1], but their importance extends beyond disease prevention in the vaccinated. By contributing to herd immunity, they can interrupt chains of transmission and reduce disease in those who are not vaccinated. This can lead to a reduction in healthcare usage, preserve workforce productivity and protect the vulnerable from exposure. However, with all vaccines, there is variability in the effectiveness and durability of protection, with the most significant variability evident in older people. Immunosenescence is a complex process which ultimately leads to a decline in the amount and function of immune cells and impaired immune responses [2, 3]. Several strategies can be employed to optimise vaccine immunogenicity in older people [4, 5].

Previous experimental and observational work from our group and others has shown that a range of emotional and lifestyle factors affect vaccine effectiveness, including positive and negative mood [6–9]. Systematic reviews replicate these findings, with consistent support for negative psychological experiences (e.g. stress, depression) predicting weaker antibody responses, whereas positive psychosocial attributes—such as social support, positive mood—are linked with enhanced immunogenicity following vaccination [10, 11]. These effects may occur via neuroimmune and hypothalamic-pituitary axis pathways directly altering immune function and/or indirectly through health behaviours, such as increased physical activity.

Nutritional determinants of immune responses have also been the subject of much interest [12], with vitamin D among those to have received particular attention due to its key role in immune regulation [13]. Many immune cells express vitamin D receptors, and experimental studies demonstrate direct effects on immune parameters [14]. Vitamin D deficiency has been associated with poorer vaccine responses in epidemiological studies [15].

The challenge from a population health perspective is whether and how this evidence can be translated to realise clinical benefit. In this regard, recent work has suggested that, in terms of psychological parameters, positive mood at the time of vaccination may exert a larger effect than negative mood on antibody responses to vaccination [7]. Furthermore, it is possible to boost positive mood in older adults at the time of vaccination through a brief digital intervention [8, 16]. These data point towards potential for brief interventions that enhance mood at the time of vaccination to optimise vaccine effectiveness. Similarly, despite the wide availability of vitamin D supplements without prescription, uptake among older adults is known to be low [17, 18]. Taken together, there would appear to be potential to examine the independent and combined effects of interventions to enhance mood and vitamin D on the effectiveness of vaccinations in older adults. However, we know little about the acceptability of such treatments to the target patient group or how to design a trial which maximises engagement with these interventions.

Thus, we designed a feasibility study to examine potential challenges associated with offering a brief PMI and vitamin D to older adults at the time of vaccination. This was designed as a pilot for a future randomised controlled trial to investigate the effect of a PMI, with or without vitamin D supplementation, on vaccine immunogenicity in older adults:

PROTECTIVE-D: Improving the effectiveness of vaccination with positive mood and vitamin D

### Objectives

1. Assess the feasibility of, and compliance with, vitamin D supplementation for 28 days and a brief PMI for 7 days in older adults.
2. Assess the feasibility of daily reminders to complete study procedures sent to participants in the intervention groups and the response rate.
3. Assess the feasibility and compliance with conventional venous sampling and finger-prick capillary sampling for immunological outcome assessment.
4. Gather preliminary evidence on the effects of the PMI alone, vitamin D alone and the PMI and vitamin D in combination on the antibody response to influenza vaccine.

## Methods

### Trial design

A four-arm parallel group randomised controlled feasibility trial.

### Participants

Older adults aged 65 years and over were invited to participate by their NHS general practice at which they were all registered patients. The initial contact was made by SMS text to a mobile phone with a response link to the investigator, email from the practice research mailbox or telephone call made by a member of the study team who was also a member of the practice direct care team. Potential participants who expressed an interest after this contact were sent an electronic copy of the approved Patient Information Sheet at least 24 h before their first study visit, and those willing to proceed to screening were consented by the investigator.

The influenza vaccine given to participants was the NHS seasonal vaccine for 2022/2023. Recruitment took place in May and June 2023, and all participants had previously received the same vaccine in September or October 2022 in accordance with the national schedule.

Inclusion criteria were broad to maximise the representativeness of the cohort. Therefore, exclusions were restricted to those who were unable to attend study visits (because they were coded as housebound on their medical records or because they had other commitments during the study period) or had cognitive impairment limiting their capacity to provide informed consent and perform study procedures (in the opinion of the investigator). Additionally, potential participants who were prescribed oral vitamin D supplementation or had taken it regularly on the advice of a healthcare professional were ineligible.

### Interventions

The development of the PMI (and the involvement of participants and the public) used in this study is described elsewhere [7]. In brief, it consists of a 15-min video compilation of comedy clips and uplifting music designed to appeal to older adults living in the UK. In two previous trials, it has been shown to result in significant improvements in positive mood [7, 8]. In preparation for a future trial, two main differences were introduced to the intervention. First, it was accessed via an individualised URL, sent to participants in an email, enabling participants to view the PMI at a time of their choosing and on their preferred device. In our previous studies, the PMI was viewed on a tablet in a primary care setting. Second, participants were sent a unique link every day for 7 days, with different content on each day. This was to try and achieve an increase in positive mood over a longer period, commensurate with when the primary immune response to vaccination is mounted. In our previous trials, the PMI was only implemented once on the day of vaccination.

A 28-day supply of a widely available vitamin D supplement (1000 IU) was given to participants randomised to the vitamin D supplementation condition. The dose was one per day, and a daily SMS was sent reminding these participants to take their supplement from day 1 to day 28.

Participants randomised to the usual care control group were not sent links to the PMI and were not given vitamin D supplements after receiving their influenza vaccination.

### Outcomes

The uptake of invitations to view the PMI in those randomised to this condition was determined by affirmative responses (as solicited) to the email video link sent every day from day 1 to 7 days. These links were sent manually by the investigator from a secure NHS email account.

The uptake of the vitamin D supplementation in those randomised to this condition was determined by affirmative responses (as solicited) to the SMS text reminder sent every day from day 1 for 28 days to the participant’s mobile phone. These were sent automatically using secure NHS approved software, but for technical reasons, participants did not receive the daily reminders on 2 of these days. The text of the messages was as follows:

“Thank you for being part of the PROTECTIVE-D study.

Please respond to this message to confirm you have taken your vitamin D tablet today.

You do not need to put any information in your reply. Just respond with ‘yes’ and press send.”

The measurement of antibodies to the vaccine was considered a likely primary or secondary outcome in a future trial. Thus, we also examined the feasibility of, and compliance with, finger-prick capillary sampling as a means of collecting blood samples in this age group that would permit an assessment of antibodies. This technique had been used successfully for this purpose in a younger age group in a previous study and is described elsewhere [16]. All participants were shown how to collect the sample at the day 0 visit by a clinical member of the study team and provided with a take-home self-collection kit. They were asked to perform the procedure at home just before attending their day 28 appointment. The acceptability of this technique was determined by the number of participants returning a sample at this appointment.

Although the study was not powered to detect between-group differences in antibody levels, antibody titres to three of the antigenic components of the seasonal influenza vaccine of 2022 (Influenza A/Darwin (H3N2), Influenza B/Austria and Influenza B/Phucket) were measured at day 0 (prior to vaccination) and day 28 post-vaccination on a 5 ml venous blood sample taken at the visits by a member of the study team. This allowed us to test the clinical and laboratory procedures that would need to be undertaken in a large-scale trial.

Vitamin D status was determined by an additional 5 mL venous blood sample taken at day 0 (prior to vaccination) and day 28 visits by a member of the study team. This was measured as it will be part of any future trial requiring comparison between baseline levels of serum vitamin D with those after a course of supplementation.

### Sample size

As this was a feasibility study designed to examine the acceptability of our trial procedures only, no sample size calculations were performed. We considered that a minimum target sample size of 5–6 participants per group was both pragmatic (i.e. achievable in the time-frame available for recruitment), whilst also providing a provisional estimate of likely feasibility and compliance with our trial procedures.

### Randomisation

A random allocation table was prepared by a researcher not involved in the study’s delivery. A random number generator (Random #) was used to assign participants to a place on the table. Allocation was not concealed. There were 4 conditions:

1. PMI + vitamin D supplementation
2. PMI
3. Vitamin D supplementation
4. Usual care

Allocation was in a 1:1:1:1 ratio.

### Blinding

There was no blinding in this study except for the laboratory analyses, which were carried out blind to allocation.

### Analytical methods

The 5 ml venous sample for immunology was processed and analysed in the University laboratory of the investigators. An in-house ELISA was developed to measure plasma IgG; 384-well NUNC maxisorp plates were coated with either 2 µg/mL of Influenza A/Darwin/6/2021 (IVR-227), Influenza B/Austria/1359417/2021 (BVR-26), or Influenza B/Phucket/3073/2013 (NIBSC), diluted in carbonate-bicarbonate buffer and incubated at 4 °C overnight. The plates were washed 3 times with 0.05% Tween-20 in PBS and blocked with 1% BSA (Sigma-Aldrich) in PBS for 1 h on a shaker at room temperature. The plates were washed as before and incubated with 20 μL of plasma samples diluted 1:16,000 in PBS for 2 h on a shaker, at room temperature. The plates were washed as before and then incubated with 20 μL of gamma chain-specific anti-human IgG-HRP conjugate (Sigma-Aldrich) at a 1:30,000 dilution in 1% BSA in PBS, for 1 h on a shaker at room temperature. The plates were washed as before and subsequently incubated for 20 min with 40 μL of UltraTMB substrate solution (ThermoScientific). Forty microlitres of 2 N H_2_SO_4_ was immediately added and the plate was read at 450 nm using an EPOCH microplate reader (BioTek). All assays were performed on Opentrons OT-2 liquid handling robots.

Antigen-specific IgG Absorbance (OD) values were compared between groups at day 0 and day 28. The mean intra-assay Coefficient of Variation across all strains was 4.0%.

The venous sample used for vitamin D assessment was sent to the local NHS accredited laboratory for assay. Details of how this was performed are not available.

## Results

### Participant flow

**Fig. 1:**
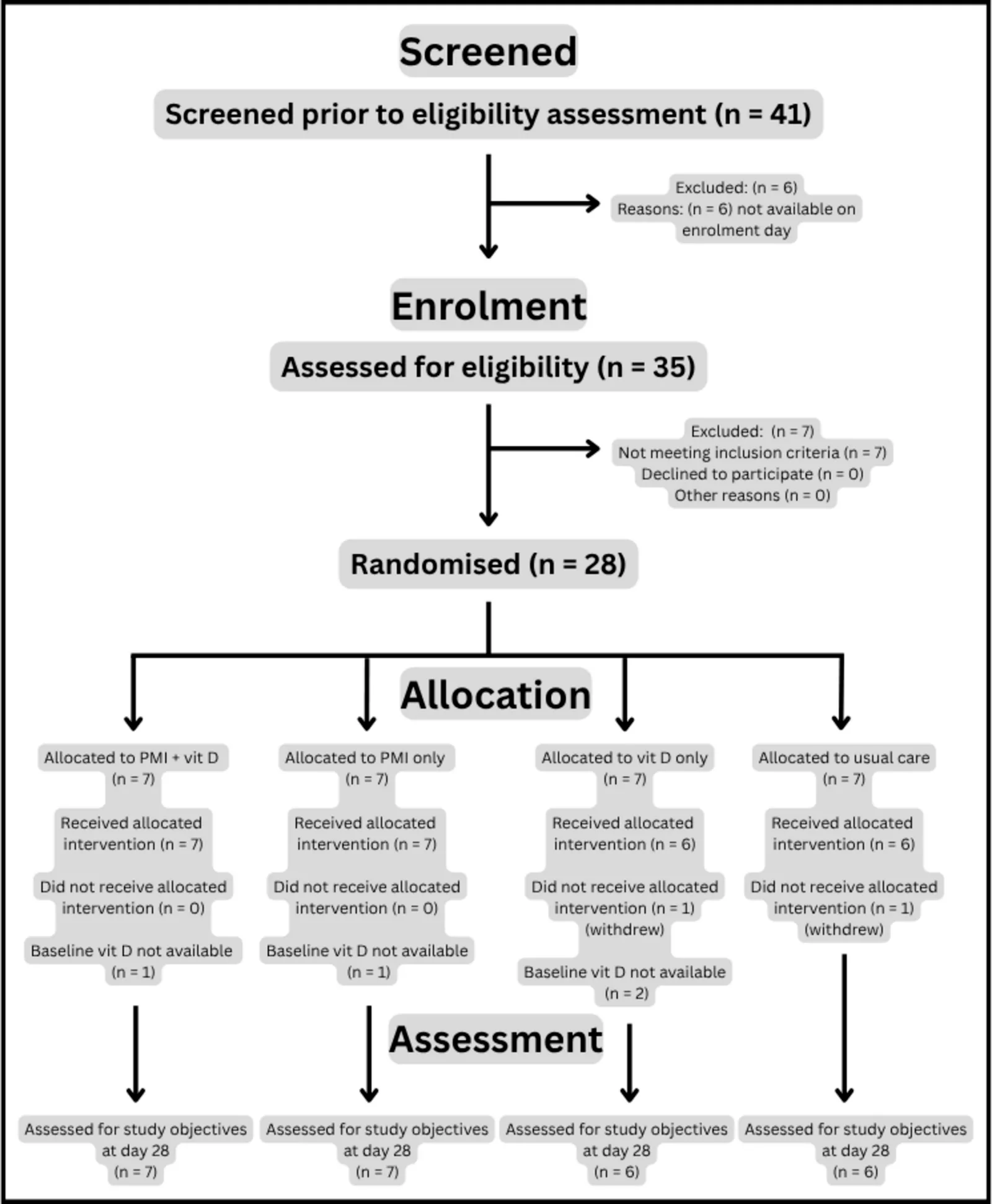
Flow diagram

Figure 1 is a flow diagram illustrating the movement of participants through the study.

### Recruitment

Forty-one potential participants were identified in a database search conducted by the investigator based on inclusion and exclusion criteria, and after screening, 35 invitations were sent. Twenty-eight participants gave informed consent and were randomised. Two participants, a husband and wife, were withdrawn after randomisation when it emerged that a pre-existing condition in one of them was worsening and it would be impossible for them to comply with further study procedures. One was allocated to usual care, and the other to vitamin D supplementation. Neither received any intervention.

### Baseline data

The average age of the 28 participants was 79.2 years (youngest 69 years, oldest 91 years). There were 12 females and 16 males. All were of white British ethnicity.

### Numbers analysed

Baseline (day 0) blood samples were not analysed in the 2 participants who were withdrawn after randomisation. A technical problem in the accredited laboratory used by the study for vitamin D analysis resulted in day 0 vitamin D levels being unavailable for 4 of the remaining 26 participants. Day 28 vitamin D level samples and immunology samples at day 0 and day 28 were analysed in all 26 remaining participants. One participant in the vitamin D supplementation condition had high levels of vitamin D at day 0 (215 nmol/l—upper limit of normal 200) and day 28 (142 nmol/l). The levels were inconsistent with the clinical history and these readings were excluded when calculating average changes in vitamin D levels.

### Outcomes

All 7 participants allocated to the PMI condition and all 7 participants allocated to the PMI + vitamin D supplementation condition reported viewing the PMI on all 7 days.

Only 1 participant out of 13 in the vitamin D supplementation condition had difficulty using the SMS response links to confirm taking the tablet. This appeared to be due to a combination of hardware problems and poor reception. Eight responded in the affirmative to all their reminders, 1 responded to 25 out of 26, 1 to 24, 1 to 23 and 1 to 19.

Sixteen out of 26 participants (62%) returned a finger-prick blood sample taken at their home on the morning they attended their day 28 appointment. The remaining 10 asked if this could be taken by a clinical member of the research team during the appointment because they did not wish to or did not feel able to take it themselves.

Visual inspection of finger-prick samples indicated some participants did not fill the mitre stick to the correct level. The finger-prick samples were not analysed.

Nine paired venous blood samples (day 0 and day 28) were obtained from participants in the vitamin D supplementation condition. The average increase was 8.6 nmol/l. Twelve paired samples were obtained from participants who did not receive vitamin D supplementation, and in these, the average increase was 1.7 nmol/l.

**Fig. 2:**
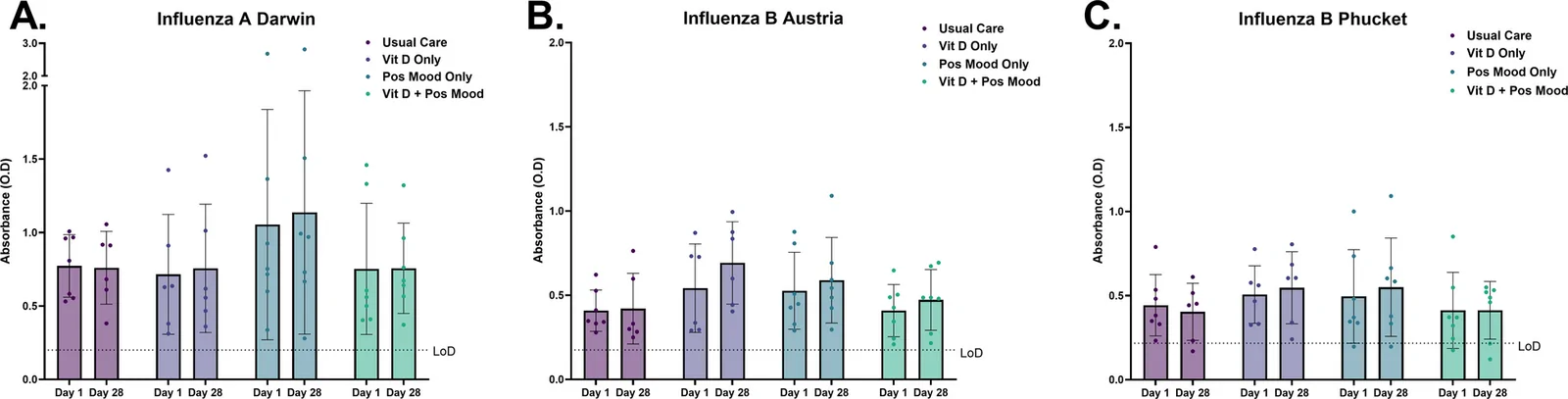
IgG titres before and 28 days after conditions. The IgG absorbance (O.D) values measured by ELISA at day 0 and day 28 of usual care, Vitamin D only, Positive mood only, or Vitamin D with positive mood. **A** Influenza A Darwin-specific IgG titres, **B** Influenza B Austria-specific IgG titres and **C** Influenza B Phucket-specific IgG titres

Changes in influenza antibody concentration measured in venous blood between day 0 and day 28 are shown in Fig. 2.

### Harms

No adverse events or harms were reported during the trial. The participant who had elevated vitamin D levels at baseline has continued to have normal vitamin D levels at subsequent annual checks.

## Discussion

This study aimed to assess the feasibility of, and compliance with, a brief PMI and vitamin D supplementation in older adults, and to evaluate the feasibility of self-collected blood sampling and electronic monitoring of compliance.

### Limitations and generalisability

This is a feasibility study, so formal hypothesis testing was not appropriate.

Participants came from a single NHS general practice in Nottingham and were not diverse in their socioeconomic grouping or ethnicity. This may negatively affect the generalisability of our findings.

Furthermore, contrary to recommendations, we did not stipulate formal progression criteria [19]. 

We have assumed that compliance with study procedures is an indicator of their acceptability to the participants. The collection of qualitative data relating to participants’ views on the procedures was not a part of this investigation.

The use of antibody titres as an indicator of vaccine effectiveness in older people is problematic. It should be seen as an imperfect proxy since factors such as virus mismatch, waning over the course of the influenza season, prior influenza exposure, frailty and co-morbidity and cell-mediated immunity can have confounding influences [20, 21].

### Interpretation

This study has shown that a brief PMI and vitamin D supplementation interventions are acceptable to older adults in this sample and could be tested feasibly in a larger randomised controlled trial. Previous studies using the PMI [7, 8] have demonstrated the effectiveness of the intervention at a single exposure, but this is the first time it has been shown to be acceptable repeatedly for seven days.

The use of SMS texts with response links appears to be acceptable to this population and may be useful in monitoring participant-reported outcomes, including compliance with study procedures. This could present a cost-effective and platform-independent alternative to traditional diaries and e-diaries. Systematic reviews support the use of SMS texts as a means of improving medication adherence in older people [22], and qualitative data suggests that alongside other means of electronic communication they are acceptable to this population [23]. 

Finger-prick capillary sampling at home was acceptable to the majority of the participants and could be incorporated into the design of a larger trial and thus limit the number of study visits, although consideration should be given to finding ways to assist participants who may find the procedure challenging. This finding is supported in the literature [24, 25]. We have previously used this technique successfully in a younger age group [16], and age-related reductions in dexterity are a possible issue to consider. Additional training, optimised instructions and remote support may assist in overcoming this obstacle.

The effect of PMI and vitamin D supplementation on antibody response to influenza vaccination is worthy of further investigation.

## Conclusion

This feasibility study has demonstrated the acceptability of the proposed methodology for a large, randomised trial in older adults. A pilot study for this with formally defined progression criteria is in preparation. Further refinement of the finger-prick capillary sampling procedure is required before this technique can be employed successfully in an older population.

## Supplementary Information

Supplementary Material 1.

## Data Availability

The datasets analysed during the current study are available from the corresponding author on reasonable request.

## Abbreviations

NHS: UK National Health Service
PMI: Positive mood intervention
RCT: Randomised controlled trial
SMS: Short Message Service
