# Supplementary material for: PROTECTIVE-D—a feasibility randomised controlled trial examining the effect of a positive mood intervention and vitamin D supplementation on the immunological responses to influenza vaccination in older adults
Source: Pilot Feasibility Stud. 2026 Jul 14;12:143. doi: 10.1186/s40814-026-01881-z (PMC13643965; doi:10.1186/s40814-026-01881-z)
Supplement: Supplementary file 1 [file 40814_2026_1881_MOESM1_ESM.docx]

# CONSORT 2010 Checklist for Pilot and Feasibility Trials

Adapted from: Eldridge SM et al. CONSORT 2010 statement: extension to randomised pilot and feasibility trials. BMJ 2016;355:i5239.

## Title and Abstract

| Checklist Item | Information Reported / Notes |
| --- | --- |
| 1a. Identify as a pilot or feasibility trial in the title. | Yes |
| 1b. Structured summary of trial design, methods, results, and conclusions. | Yes |

## Introduction

| Checklist Item | Information Reported / Notes |
| --- | --- |
| 2a. Scientific background and rationale for future definitive trial; explain why pilot trial needed. | Yes |
| 2b. Specific objectives or research questions, including feasibility objectives. | Yes |

## Methods

| Checklist Item | Information Reported / Notes |
| --- | --- |
| 3a. Describe trial design (e.g., parallel, factorial), allocation ratio. | Yes |
| 3b. Report important changes to methods after trial commencement, with reasons. | Yes |
| 4a. Eligibility criteria for participants. | Yes |
| 4b. Settings and locations where data collected. | Yes |
| 5. Interventions for each group, with sufficient detail to allow replication. | Yes |
| 6a. Prespecified assessments/measurements to address each pilot objective, including timing. | Yes |
| 6b. Changes to outcomes after commencement, with reasons. | Yes |
| 6c. If applicable, describe progression criteria for proceeding to main trial. | N/A |
| 7a. Rationale for sample size determination. | N/A |
| 7b. Interim analyses and stopping guidelines (if relevant). | N/A |
| 8a. Method of generating allocation sequence. | Yes |
| 8b. Type of randomization and details of any restriction. | Yes |
| 9. Allocation concealment mechanism. | N/A |
| 10. Implementation (who generated sequence, enrolled participants, assigned interventions). | Yes |
| 11a. Blinding: who was blinded and how. | Yes |
| 11b. Similarity of interventions (if relevant). |  |
| 12. Statistical methods used to address each pilot objective. | N/A |

## Results

| Checklist Item | Information Reported / Notes |
| --- | --- |
| 13a. Participant flow: numbers assigned, received treatment, analysed. | Yes |
| 13b. Losses and exclusions after randomization, with reasons. | Yes |
| 14a. Recruitment and follow-up dates. | Yes |
| 14b. Why trial ended or stopped. | Yes |
| 15. Baseline demographic and clinical characteristics. | Yes |
| 16. Numbers analysed for each objective. | Yes |
| 17. Results for each objective, with estimates of uncertainty (e.g., 95% CI). | N/A |
| 18. Ancillary analyses (if any). | N/A |
| 19. Harms or unintended effects. | Yes |

## Discussion

| Checklist Item | Information Reported / Notes |
| --- | --- |
| 20. Limitations of the pilot trial (e.g., potential bias, imprecision). | Yes |
| 21. Generalizability of pilot trial methods and findings to future definitive trial. | Yes |
| 22. Interpretation in light of pilot objectives; implications for progression to main trial. | Yes |

## Other Information

| Checklist Item | Information Reported / Notes |
| --- | --- |
| 23. Trial registration (name, number). | Yes |
| 24. Access to full trial protocol. | Yes |
| 25. Sources of funding and role of funders. | Yes |
